# Effects of climate warming and human activities on the distribution patterns of *Fritillaria unibracteata* in eastern Qinghai-Tibetan Plateau

**DOI:** 10.1038/s41598-023-42988-0

**Published:** 2023-09-22

**Authors:** Dan Zhao, Jun Wang, Wei Dai, KunHao Ye, Jie Chen, Qianglong Lai, Haiying Li, Binglian Zhong, Xiaoli Yu

**Affiliations:** 1 Crop Characteristic Resources Creation and Utilization Key Laboratory of Sichuan Province, Mianyang Academy of Agricultural Sciences, Mianyang, 621023 People’s Republic of China; 2 College of Life Science and Biotechnology, Mianyang Normal University, Mianyang, 621000 People’s Republic of China

**Keywords:** Climate change, Climate-change ecology

## Abstract

*Fritillaria unibracteata* is an endangered medicinal material species endemic to the Qinghai Tibet Plateau, and belongs to the national Class III endangered plant. In addition to expelling wind and removing damne, it also warms menstruation and relieves pain in clinic use of tranditional Chinese medicine. In recent years, affected by the destruction of shrubs and climate change, the habitat of *F.* *unibracteata* wild resources has been seriously damaged, indicating of great significance to predict its potential suitable habitat using MaxEnt model. The AUC values without human activities were 0.983 ± 0.013–0.988 ± 0.001, while it is 0.982 ± 0.015–0.989 ± 0.000 with human activities, justifying their applications for predicting the potential areas of *F.* *unibracteata.* Without human activities, there were 8.47 × 10^4^ km^2^ of highly suitable habitats in northern Sichuan, southern Gansu and southeastern Qinghai. But the poorly, moderately and highly suitable areas of *F.* *unibracteata* have decreased to 33.8 × 10^4^ km^2^, 9.66 × 10^4^ km^2^ and 6.64 × 10^4^ km^2^ due to human activities. Environmental variables affecting *F. unibracteata* distribution included the minimum temperature in the coldest month (−16.89–−4.96 °C), annual precipitation (416.64–866.96 mm), temperature annual range (24.83–31.97 °C), elevation (2879.69–3981.82 m), human footprint (2.58–23.66) and mean UV-B of highest month (7381.92–8574.27 kJ/m^2^). In the 2050s and 2090s, human activities would significantly reduce the highly suitable habitats of *F. unibracteata*. Under SSP1-2.6, SSP2-4.5 and SSP5-8.5 scenarios, the centroid would move to the low latitude area from the current position first, and then to a high latitude area. Wild resources of *F. unibracteata* in China can be effectively conserved based on our results.

## Introduction

In the past century, due to the impact of changes in natural conditions and human activities, the global climate is developing towards a warming trend^1^. In the past century, the global average temperature has increased by 0.74 °C, and extreme weather events have occurred frequently^2^. Climate change will promote the migration of species in suitable areas, and even put some species at risk of extinction^3,4^. China is extremely vulnerable to climate change with its location in the East Asian monsoon region. In recent years, researchers in China have been increasingly interested in how will climate change affects species survival and the suitable areas^4–7^.

With the intensification, generalization, and diversification of the impact of human activities on ecosystems, the spatiotemporal distribution of species has become increasingly intense and complex. The focus of ecosystem dynamics research has rapidly shifted to the patterns and mechanisms of ecosystem changes under human activity interference. Studies have confirmed that human activities have a serious impact on plant diversity^8^. Sayit et al. used MaxEnt to develop the models of predicting suitable habitats of *Calligonum mongolicum* under different climatic conditions and human activities so as to quantitatively demonstrate the different climate change scenarios^5^. In the study of invasive alien plants, Sayit et al. also chose to introduce human activities as an environmental variable, applied MaxEnt and ArcGIS to construct a suitable habitat prediction model for *Solanum rostratum*^9^. Different human activity ranges, ways, and intensities can lead to different types of land use (arable land, forest land, shrubland land, and residential land), which directly affects the spatial distribution and diversity of plants^10,11^. Therefore, taking human activities as prediction variables can more accurately show the potential distribution range and spatial pattern of species in the future.

MaxEnt (the maximum entropy model) invented by Jaynes in 1957, can infer incomplete information, and was used to predict species distributions in 2004. In today's world, MaxEnt has been widely applied in a wide range of fields, including ecology, evolution, and resource management^12,13^. MaxEnt was more accurate than other niche models, including CLIMEX, GARP, Bioclim and Domain. In spite of incomplete data on species distribution and environmental variables, it can accurately predict distribution areas of species^14^. Additionally, MaxEnt has high stability and is applied in the field of animal and plant conservation, including predicting potential habitats for medicinal plants and endangered species^15,16^. She et al. predicted the distribution hot spots of *Notopterygium incisum*, an important medicinal resource and endangered species in the Three Rivers Source Area under the future climate change scenarios using MaxEnt^17^. Ji et al.^18^ applied MaxEnt and combined with statistical methods to simulate the current and future distribution of *Paris verticillata*, explored the relationship between its geographical distribution, and understood the development trend of its geographical distribution in the future.

**Figure 1:**
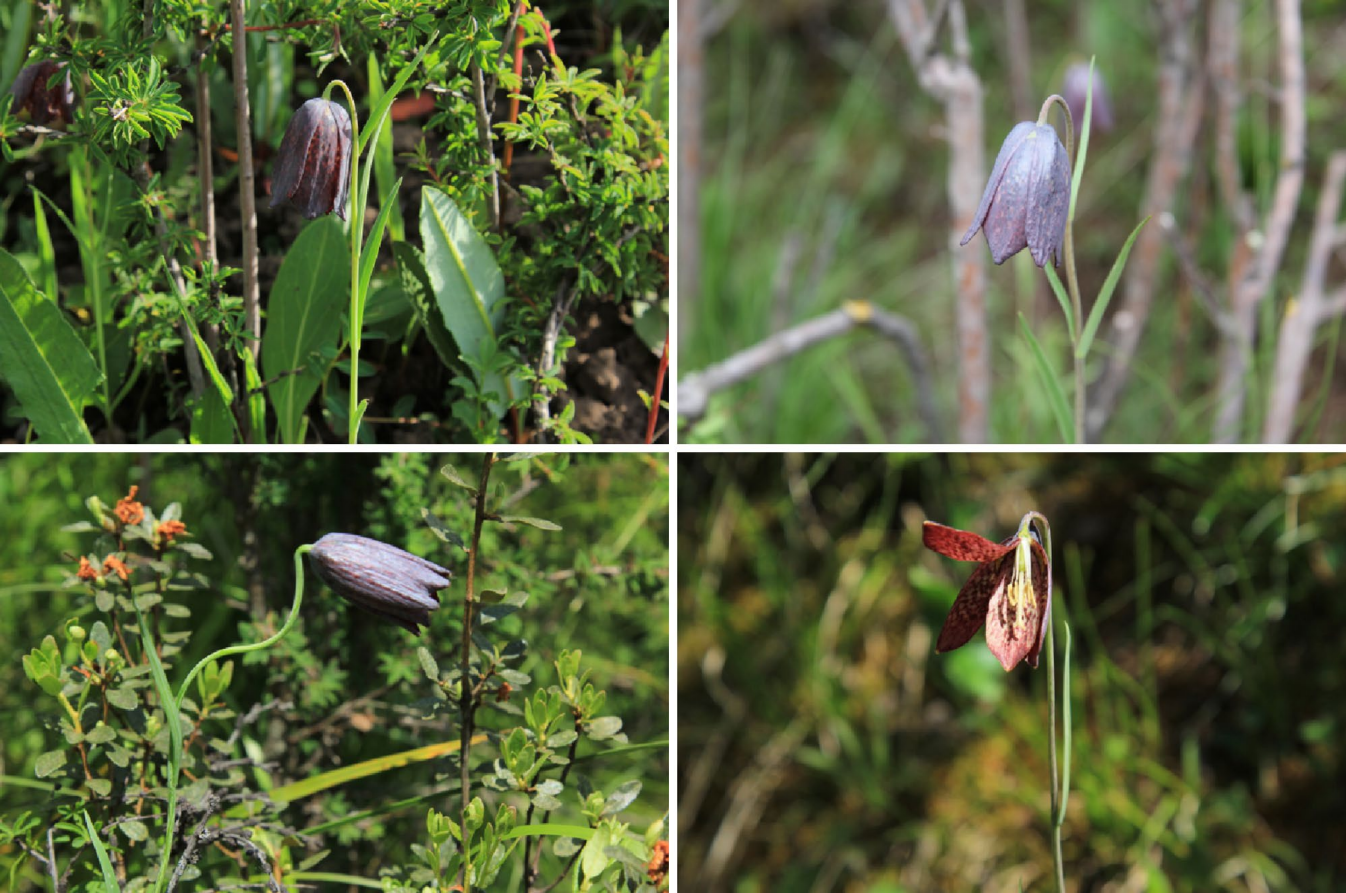
Habitat of wild *F.* *unibracteata*.

*Fritillaria unibracteata* P.K.Hsiao & K.C.Hsia (Liliaceae: *Fritillaria*) is a perennial herb (Fig. 1). The dried bulbs of *F.* *unibracteata* with important medicinal value are widely used to treat lung heat and dry cough, dry cough with less phlegm, yin deficiency and labor cough, expectoration with blood^19^. In addition, it can also reduce the permeability of blood vessels and effectively achieve the anti-inflammatory effect^19,20^. The total saponins and total alkaloids in bulbs have a certain antihypertensive effect on the cardiovascular system^19,21^. There is a growing market demand for *F. unibracteata* since it has better efficacy than other basic species of Chuanbei, such as *Fritillaria przewalskii*, *Fritillaria cirrhosa* and *Fritillaria delavayi*. As a medicinal plant, the previous research on *F.* *unibracteata* not only involves its biological characteristics, chemical components and pharmacological effects, but also involves its community ecology. Huang et al.^22^ found that the distribution of *F.* *unibracteata* is in the transition from China-Japan forest plant subregion to China-Himalayan plant subregion, which is the most abundant area of alpine flora in the world. Due to the narrow niche and long growth cycle, as well as the deterioration of the ecological environment caused by climate change and people's excessive excavation, the wild resources of *F.* *unibracteata* have been greatly reduced in recent years and have been listed as a national third-class endangered medicinal material species^22^. Currently, studies using models to simulate the habitat of *F.* *unibracteata* have not included human activities as an important variable. Wang et al. analyzed the suitable habitat of *F.* *unibracteata* in China by using TCMGIS-I system^23^. Two reports have applied MaxEnt model to simulate the distribution of *F.* *unibracteata* with the impact of current climate condition^24,25^. On the basis of previous studies, we introduced human activities as a comparative variable, and our purpose was to solve the following issue: (1) analyze the key environmental variables affecting the potential distribution of *F.* *unibracteata*. (2) simulate the distribution pattern of *F.* *unibracteata* in eastern Qinghai-Tibetan Plateau. (3) clarify the role of human activities in the geographical distribution of *F.* *unibracteata*. The results can provide data support for the wild cultivation and introduction of *F. unibracteata*, and provide theoretical basis for the protection and scientific research of the Fritillaria genus.

## Results

### Importance of environmental variables

**Figure 2:**
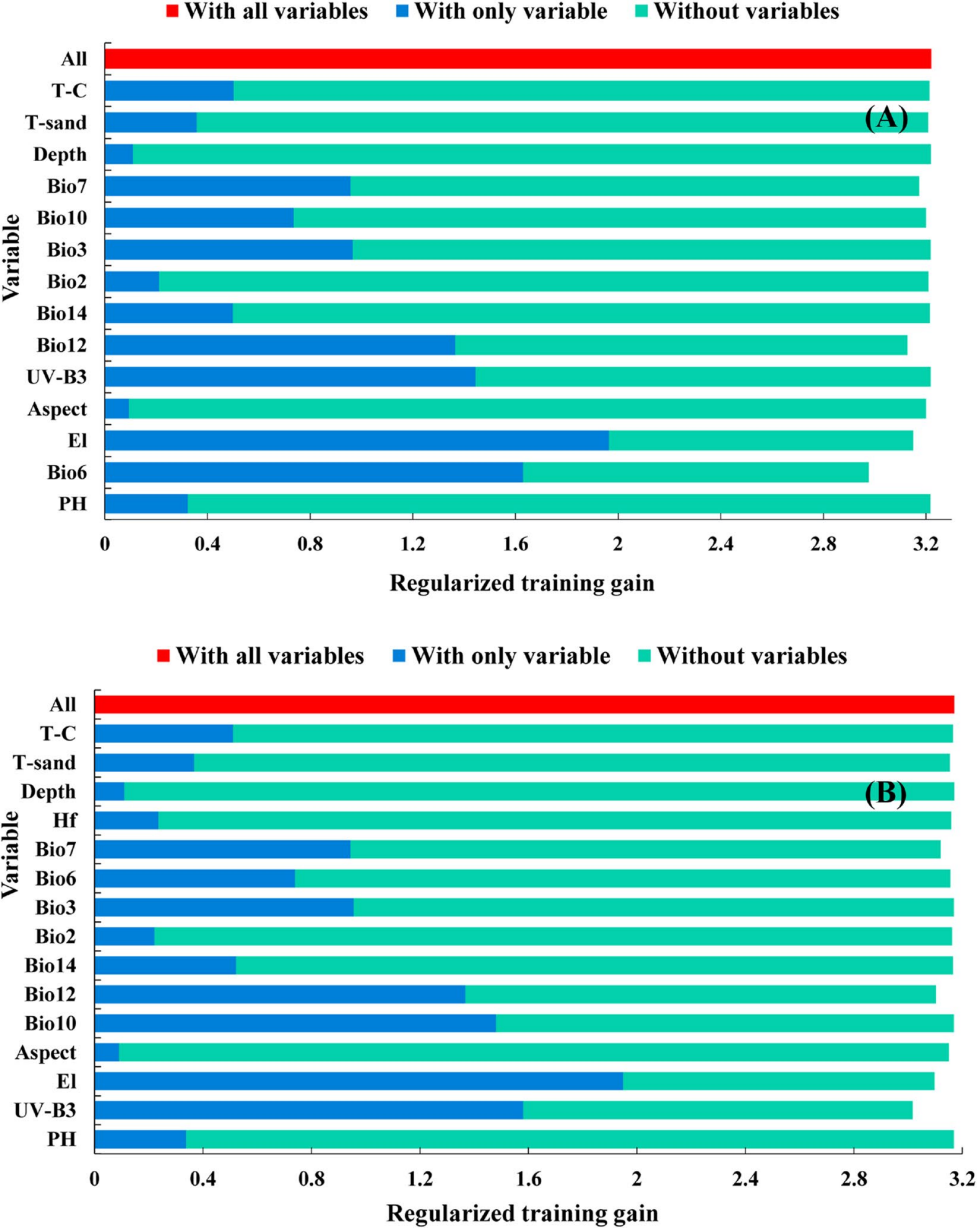
Import of environmental variables for prediction based on jackknife test.

Without human activities, the percent contribution rate (44.54%) and the regularized training gain (1.96) of elevation (El) were the highest (Fig. 2A and Table 1), indicating that it was the most important variable for the distribution of *F.* *unibracteata*. When modelling with only variable, the gain (1.63) of min temperature of coldest month (Bio6) ranked second (Fig. 2A), while modelling without Bio6, the score of the model decreased the most, indicating the influence of this variable on simulation. The training gain of mean UV-B of highest month (UV-B3) and annual precipitation (Bio12) exceed 1.3 (Fig. 2A), and their percent contribution rates reached 18.17% and 28.46% respectively (Table 1).

**Table 1:** Percent contribution of 15 environmental variables.

Variable	Percent contribution (%)	
	Without human activities	With human activities
Elevation	44.54	45.63
Annual precipitation	28.46	25.82
Mean UV-B of highest month	18.17	15.04
Temperature annual range	3.69	4.10
Potential of hydrogen	1.25	0.94
Min temperature of coldest month	1.04	1.01
Aspect	0.82	0.81
Topsoil sand fraction	0.78	1.65
Isothermality	0.42	0.40
Mean diurnal range	0.26	0.16
Topsoil sand fraction	0.20	0.21
Precipitation of warmest quarter	0.20	0.19
Reference soil depth	0.17	0.39
Mean temperature of warmest quarter	0.02	0.03
Human footprint index	/	3.61

With human activities, the percent contribution rate (45.63%) and the regularized training gain (1.95) of elevation (El) were the highest (Fig. 2B and Table 1), indicating its importance. The regularized training gain (1.58) and the percent contribution rate (15.04%) of mean UV-B of highest month (UV-B3) ranked second and third respectively (Fig. 2B and Table 1). The training gain value for "without variables" was most affected by mean UV-B of the highest month (UV-B3) (Fig. 2B), indicating that its unique information and great impact to the distribution of *F.* *unibracteata.* The regularized training gain of human footprint index was 1.48 (Fig. 2B), which showed the necessity of introducing it.

By the above comparison method, min temperature of coldest month (Bio6), elevation (El), annual precipitation (Bio12), human footprint index (Hf), mean UV-B of highest month (UV-B3) and temperature annual range (Bio7) were identified to be the dominant environmental variables.

### Suitable value range of main environmental variables

**Figure 3:**
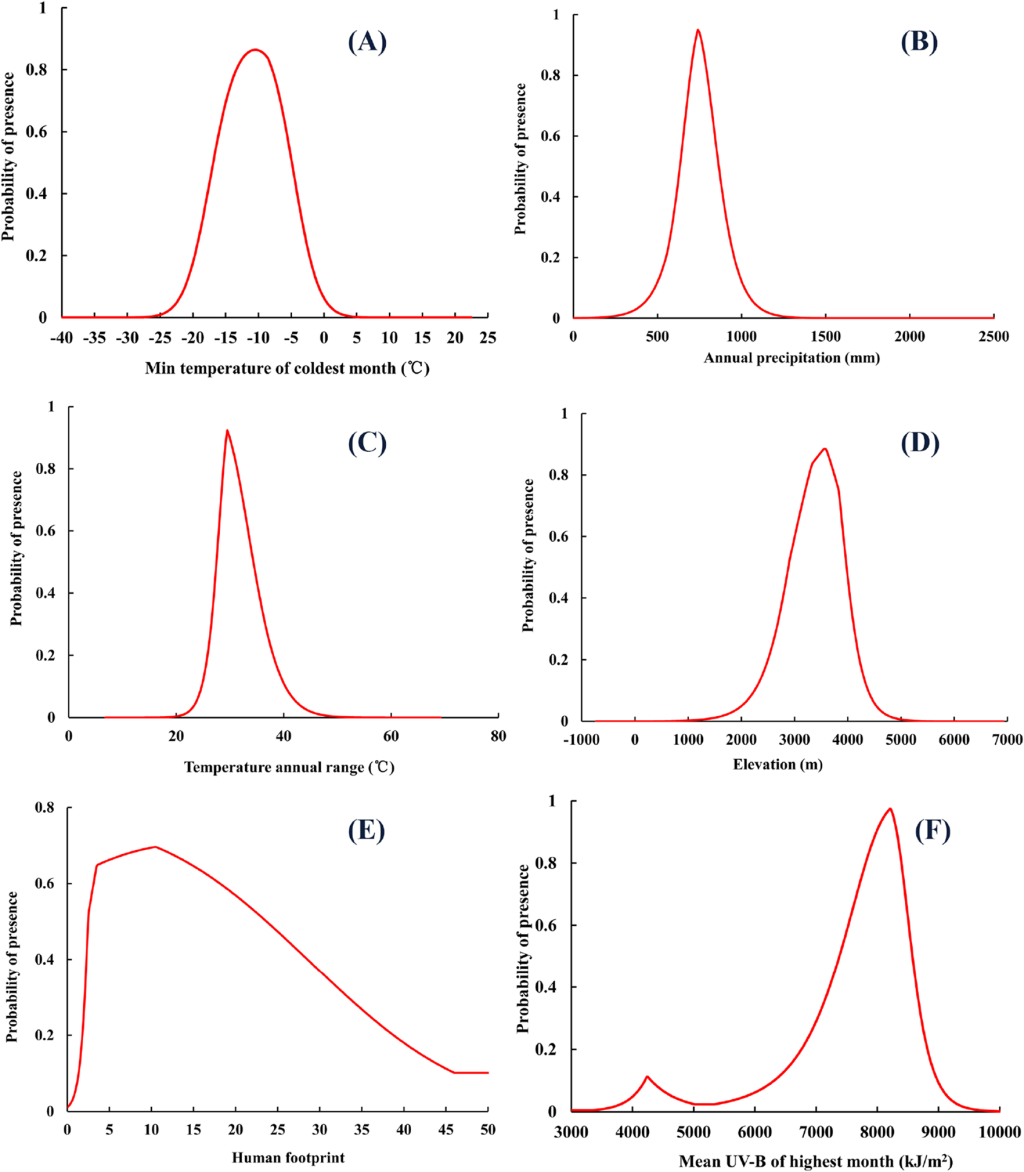
Response curves of variables.

When the elevation (El) < 3568.52 m, it had a positive impact on the presence probability of *F.* *unibracteata*, that was, the presence probability increased with elevation, while when the elevation > 3568.52 m, it had a negative impact on the presence probability (Fig. 3). When the min temperature of coldest month (Bio6) was −10.5 °C, the presence probability of *F. unibracteata* decreased steadily after reaching the peak (Fig. 3). The presence probability of *F. unibracteata* increased rapidly to the highest value 10.52 (P = 0.7) when the human footprint value reached 2.47, then followed by a decrease in probability. According to Fig. 3, the suitable ranges of min temperature of coldest month, annual precipitation, temperature annual range, elevation, human footprint and mean UV-B of highest month were −16.89–−4.96 ℃ (Fig. 3A), 416.64–866.96 mm (Fig. 3B), 24.83–31.97 °C (Fig. 3C), 2879.69–3981.82 m (Fig. 3D), 2.58–23.66 (Fig. 3E) and 7381.92–8574.27 kJ/m^2^ (Fig. 3F), respectively.

### Geographical distribution and GAP analysis of ***F. unibracteata*** under current situation

**Figure 4:**
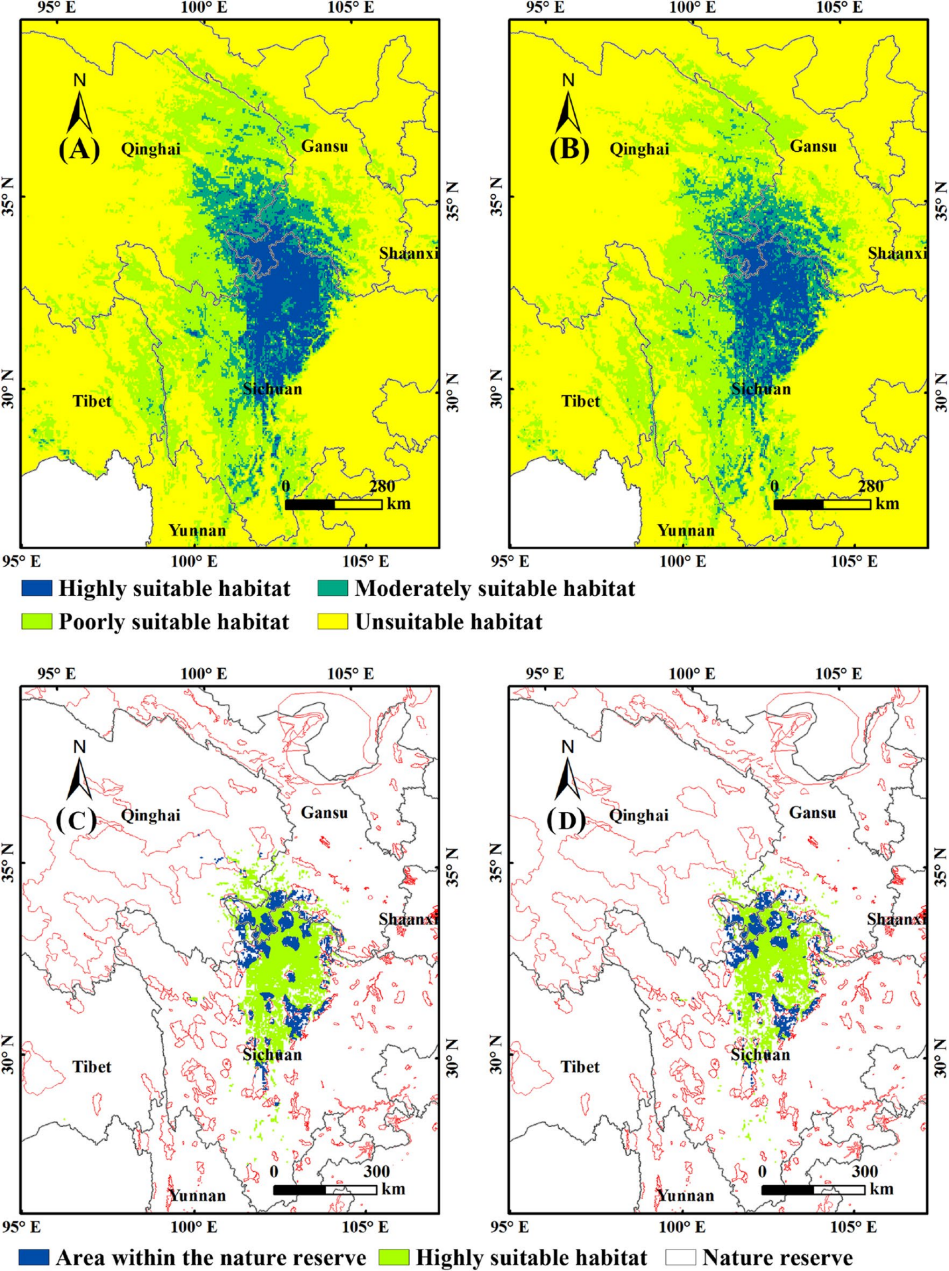
Potential distribution and GAP analysis of *F.* *unibracteata* under current condition. (**A**): without human activities; (**B**): with human activities; (**C**): without human activities; (**D**): with human activities. MaxEnt v3.4.4: https://biodiversityinformatics.amnh.org/open_source/maxent/, ArcGIS v10.0: https://www.arcgis.com/.

Without human activities, the suitable range of *F.* *unibracteata* was centered on the highly suitable habitat, and moderately and poorly suitable habitats expanded outward (Fig. 4A, Table 2). The highly suitable habitats (8.47 × 10^4^ km^2^) were distributed in northern Sichuan (6.45 × 10^4^ km^2^), southern Gansu (1.35 × 10^4^ km^2^) and southeastern Qinghai (0.67 × 10^4^ km^2^) in the eastern Qinghai Tibet Plateau. The moderately suitable habitats (10.43 × 10^4^ km^2^) extended to the periphery along the highly suitable habitats (4.26 × 10^4^ km^2^), mainly located in eastern Qinghai (3.58 × 10^4^ km^2^) and southeastern Gansu (2.24 × 10^4^ km^2^) (Fig. 4A and Table 2). The distribution of the poorly suitable habitats were the widest and scattered, including central and southern Gansu (5.53 × 10^4^ km^2^), eastern Qinghai (11.22 × 10^4^ km^2^), western and southern Sichuan (11.48 × 10^4^ km^2^), and eastern Tibet (4.49 × 10^4^ km^2^) (Fig. 4A and Table 2).

**Table 2:** The areas of suitable habitats and their changes under future climate change scenarios.

	Scenario	Area of suitable habitats (× 10^4^km^2^)				Changed area compared to current (× 10^4^km^2^)		
		Poorly	Moderately	Highly	Total	Stable	Shrink	Expand
Without human activities	Current	38.44	10.43	8.47	57.34	/	/	/
	2050s, SSP1-2.6	36.86	9.17	6.71	52.74	14.45	4.40	1.24
	2050s, SSP2-4.5	40.94	10.61	6.96	58.51	15.59	3.27	1.72
	2050s, SSP5-8.5	44.79	8.91	7.24	60.94	14.11	4.75	1.83
	2090s, SSP1-2.6	42.01	11.87	8.17	62.05	13.92	4.95	5.72
	2090s, SSP2-4.5	48.57	14.82	9.15	72.54	15.05	3.82	8.55
	2090s, SSP5-8.5	43.30	12.06	7.69	63.05	13.86	5.00	5.55
With human activities	Current	33.80	9.66	6.64	50.1	10.51	5.62	0.62
	2050s, SSP1-2.6	26.61	6.84	4.47	37.92	10.82	5.31	0.68
	2050s, SSP2-4.5	24.51	11.58	4.45	40.54	11.43	4.70	1.41
	2050s, SSP5-8.5	35.33	8.66	4.51	48.5	11.49	4.65	3.74
	2090s, SSP1-2.6	37.70	10.45	5.28	53.43	11.85	4.29	4.91
	2090s, SSP2-4.5	38.53	11.59	5.74	55.86	10.87	5.26	2.96
	2090s, SSP5-8.5	36.14	9.00	5.22	50.36	10.51	5.62	0.62

Under the influence of human activities, highly, moderately and poorly suitability areas of *F. unibracteata* were 6.64 × 10^4^ km^2^, 9.66 × 10^4^ km^2^ and 33.8 × 10^4^ km^2^, respectively (Fig. 4B and Table 2). The fragmentation of suitable habitats of *F. unibracteata* were more apparent under human activities. The areas of highly, moderately, and poorly suitable habitats decreased by 21.63%, 48.91%, and 30.84%, suggesting a negative correlation between the *F.* *unibracteata’*s distribution and human activities. GAP analysis showed that the highly suitable habitats of *F.* *unibracteata* overlapping with the nature reserve was 2.34 × 10^4^ km^2^ without human activities (Fig. 4C). With human activities (Fig. 4D), the overlapping area was 1.84 × 10^4^ km^2^, accounting for 27.71% of the highly suitable habitats.

### Future potential distribution*** of F. unibracteata***

**Figure 5:**
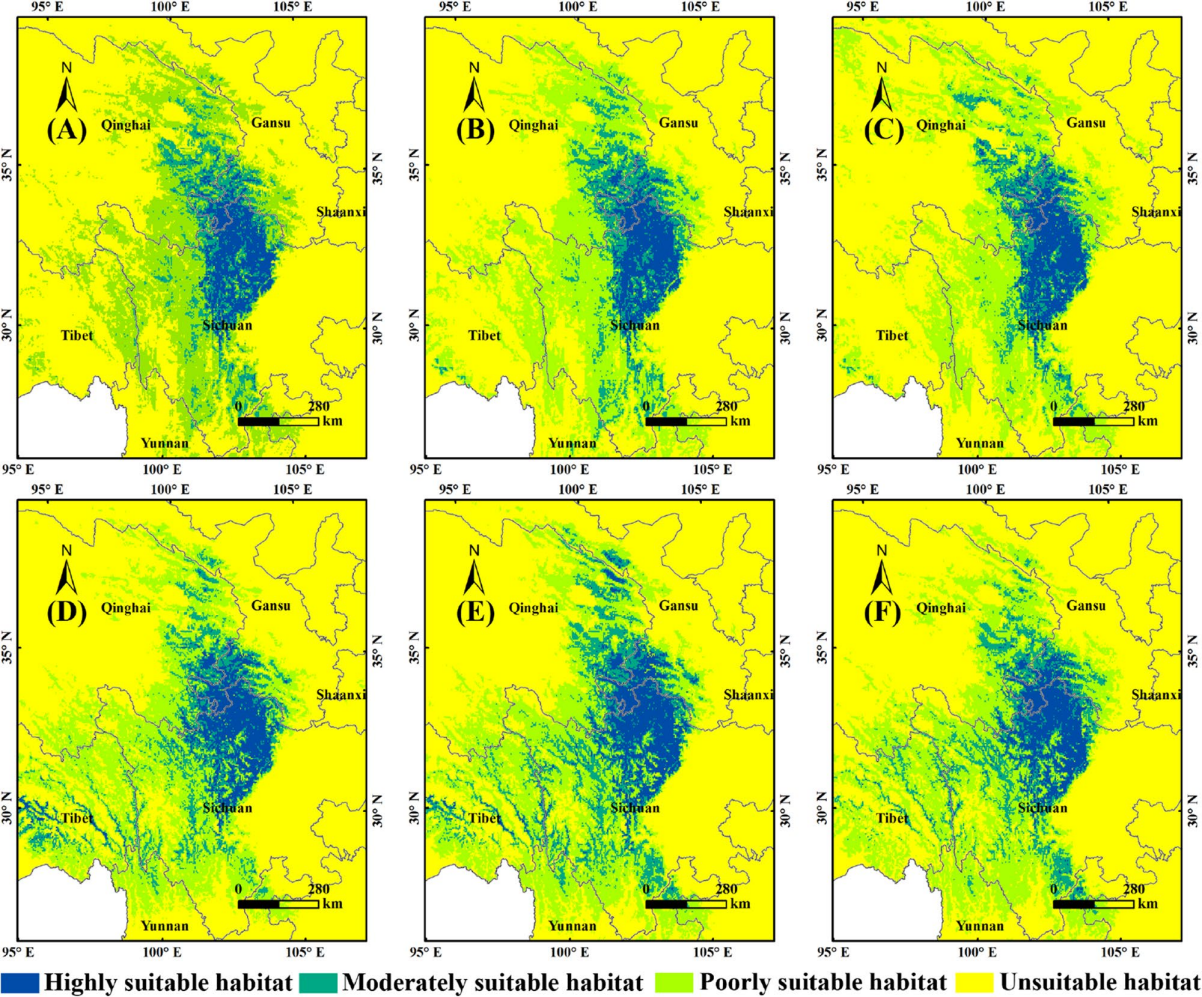
Potential distribution of *F.* *unibracteata* in the future without human activities. (**A**): 2050s, SSP1-2.6; (**B**): 2050s, SSP2-4.5; (**C**): 2050s, SSP5-8.5; (**D**): 2090s, SSP1-2.6; (**E**): 2090s, SSP2-4.5; (**F**): 2090s, SSP5-8.5. MaxEnt v3.4.4: https://biodiversityinformatics.amnh.org/open_source/maxent/, ArcGIS v10.0: https://www.arcgis.com/.

Figure 5 showed the future suitable habitats of *F.* *unibracteata* without human activities. By 2050s, the highly suitable habitats area would decrease from 8.47 × 10^4^ km^2^ to 6.71 × 10^4^ km^2^ (SSP1-2.6), 6.96 × 10^4^ km^2^ (SSP2-4.5) and 7.24 × 10^4^ km^2^ (SSP5-8.5) (Table 2). The moderately suitable habitats would decrease from 10.43 × 10^4^ km^2^ to 9.17 × 10^4^ km^2^ (SSP1-2.6) and 8.91 × 10^4^ km^2^ (SSP5-8.5), while would increase to 10.61 × 10^4^ km^2^ under SSP2-4.5 (Table 2). The poorly suitable habitats would increase from 38.44 × 10^4^ km^2^ to 40.94 × 10^4^ km^2^ (SSP2-4.5) and 44.79 × 10^4^ km^2^ (SSP5-8.5), while would decrease to 36.86 × 10^4^ km^2^ under SSP1-2.6 (Table 2). By 2090s, the highly suitable habitats would decrease to 8.17 × 10^4^ km^2^ (SSP1-2.6) and 7.69 × 10^4^ km^2^ (SSP5-8.5), while would increase to 9.15 × 10^4^ km^2^ under SSP2-4.5) (Table 2). The moderately suitable habitats would increase to 11.87 × 10^4^ km^2^ (SSP1-2.6), 14.82 × 10^4^ km^2^ (SSP2-4.5) and 12.06 × 10^4^ km^2^ (SSP5-8.5) (Table 2). The poorly suitable habitats would increase to 42.01 × 10^4^ km^2^ (SSP1-2.6), 48.57 × 10^4^ km^2^ (SSP2-4.5) and 43.3 × 10^4^ km^2^ (SSP5-8.5) (Table 2).

**Figure 6:**
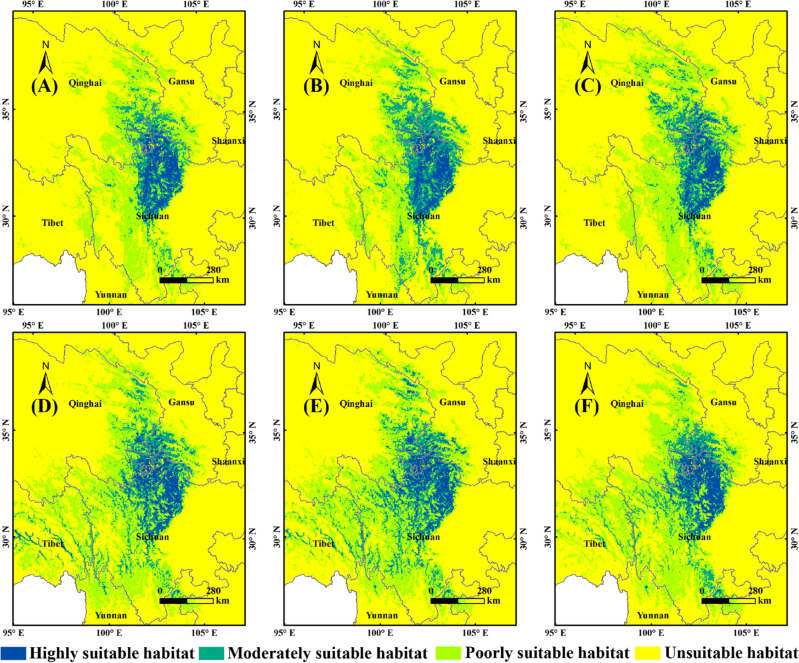
Potential distribution of *F.* *unibracteata* in the future with human activities. (**A**): 2050s, SSP1-2.6; (**B**): 2050s, SSP2-4.5; (**C**): 2050s, SSP5-8.5; (**D**): 2090s, SSP1-2.6; (**E**): 2090s, SSP2-4.5; (**F**): 2090s, SSP5-8.5. MaxEnt v3.4.4: https://biodiversityinformatics.amnh.org/open_source/maxent/, ArcGIS v10.0: https://www.arcgis.com/.

Figure 6 showed the future suitable habitats of *F.* *unibracteata* with human activities. By 2050s, the highly suitable habitats area would decrease from 6.64 × 10^4^ km^2^ (Current) to 4.47 × 10^4^ km^2^ (SSP1-2.6), 4.45 × 10^4^ km^2^ (SSP2-4.5) and 4.51 × 10^4^ km^2^ (SSP5-8.5) (Table 2). The moderately suitable habitats would decrease from 9.66 × 10^4^ km^2^ (Current) to 6.84 × 10^4^ km^2^ (SSP1-2.6) and 8.66 × 10^4^ km^2^ (SSP5-8.5), while would increase to 11.58 × 10^4^ km^2^ under SSP2-4.5 (Table 2). The poorly suitable habitats would decrease from 33.8 × 10^4^ km^2^ (Current) to 26.6 × 10^4^ km^2^ (SSP1-2.6) and 24.51 × 10^4^ km^2^ (SSP2-4.5), while would increase to 35.33 × 10^4^ km^2^ under SSP5-8.5 (Table 2). By 2090s, the highly suitable habitats area would decrease to 5.28 × 10^4^ km^2^ (SSP1-2.6), 5.74 × 10^4^ km^2^ (SSP2-4.5) and 5.22 × 10^4^ km^2^ (SSP5-8.5) (Table 2). The moderately suitable habitats would increase to 10.45 × 10^4^ km^2^ (SSP1-2.6) and 11.59 × 10^4^ km^2^ (SSP2-4.5), while decrease to 9 × 10^4^ km^2^ under SSP5-8.5 (Table 2). The poorly suitable habitats would increase to 37.7 × 10^4^ km^2^ (SSP1-2.6), 38.53 × 10^4^ km^2^ (SSP2-4.5) and 36.14 × 10^4^ km^2^ (SSP5-8.5) (Table 2).

### Changes in distribution of suitable habitats in the future

**Figure 7:**
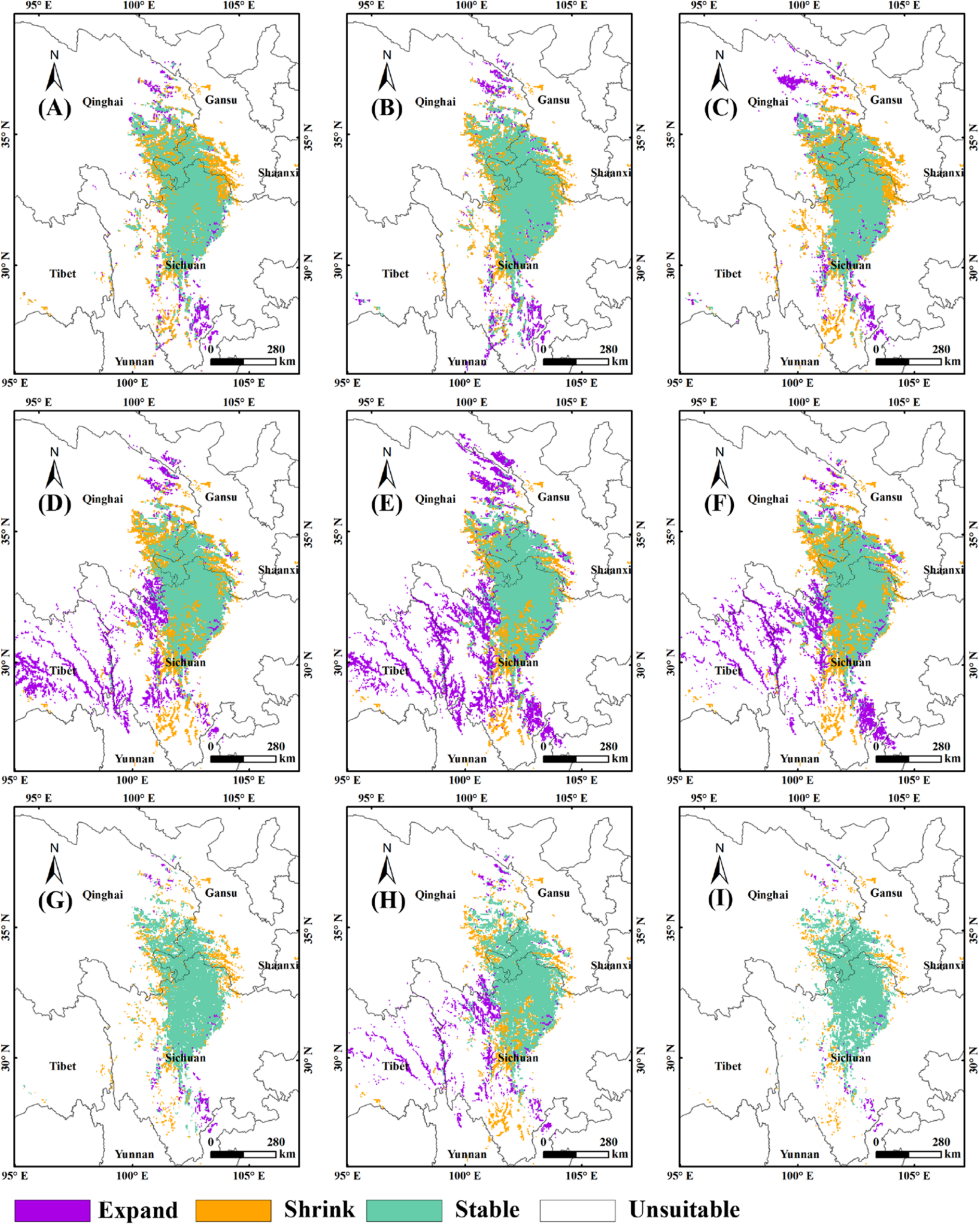
Changes in distribution of suitable habitats in the future without human activities. (**A**): 2050s, SSP1-2.6; (**B**): 2050s, SSP2-4.5; (**C**): 2050s, SSP5-8.5; (**D**): 2090s, SSP1-2.6; (**E**): 2090s, SSP2-4.5; (**F**): 2090s, SSP5-8.5. MaxEnt v3.4.4: https://biodiversityinformatics.amnh.org/open_source/maxent/, ArcGIS v10.0: https://www.arcgis.com/.

Figure 7 showed the stable, expand and shrink area of the suitable habitats in 2050s and 2090s without human activities compared with current situation. In 2050s, the proportion of the stable habitats to the current was 76.65% (SSP1-2.6), 82.65% (SSP2-4.5) and 74.8% (SSP5-8.5) respectively (Fig. 7A,B,C), while in 2090s it was 73.78% (SSP1-2.6), 79.75% (SSP2-4.5) and 73.49% (SSP5-8.5) respectively (Fig. 7D,E,F). The proportion of the shrink habitats to the current was 23.35% (SSP1-2.6), 17.35% (SSP2-4.5) and 25.2% (SSP5-8.5) respectively in 2050s (Fig. 7A,B,C), and 26.22% (SSP1-2.6), 20.25% (SSP2-4.5) and 26.51% (SSP5-8.5) in 2090s (Fig. 7D,E,F). The proportion of the expand habitats to the future was 7.91% (SSP1-2.6), 9.95% (SSP2-4.5) and 11.48% (SSP5-8.5) respectively in 2050s (Fig. 7A,B,C), and 29.14% (SSP1-2.6), 36.23% (SSP2-4.5) and 28.6% (SSP5-8.5) in 2090s respectively (Fig. 7D,E,F). By overlaying layers in ArcGIS, the suitability changes of the suitable habitats in 2050s and 2090s were obtained (Fig. 7G,H). The results showed that Aba, Ruoergai, Hongyuan, Songpan, Markang, Heishui, Jinchuan, Lixian, Maoxian, Xiaojin, Danba, Wenchuan, Baoxing, Kangding and Luding in the western Sichuan Plateau, Maqu, Luqu, Xiahe and Hezuo in the western Gannan Plateau, Henan, Zeku, Tongren and Jiuzhi in the eastern Qinghai were identified as stable habitats for the growth and distribution of *F.* *unibracteata* (Fig. 7G,H)*.* The expand habitats were distributed in the western Sichuan Plateau, the east of Tibet, and the northeast of Qinghai Province in strip or point form (Fig. 7G). The shrink habitat was scattered around the stable area (Fig. 7G,H).

**Figure 8:**
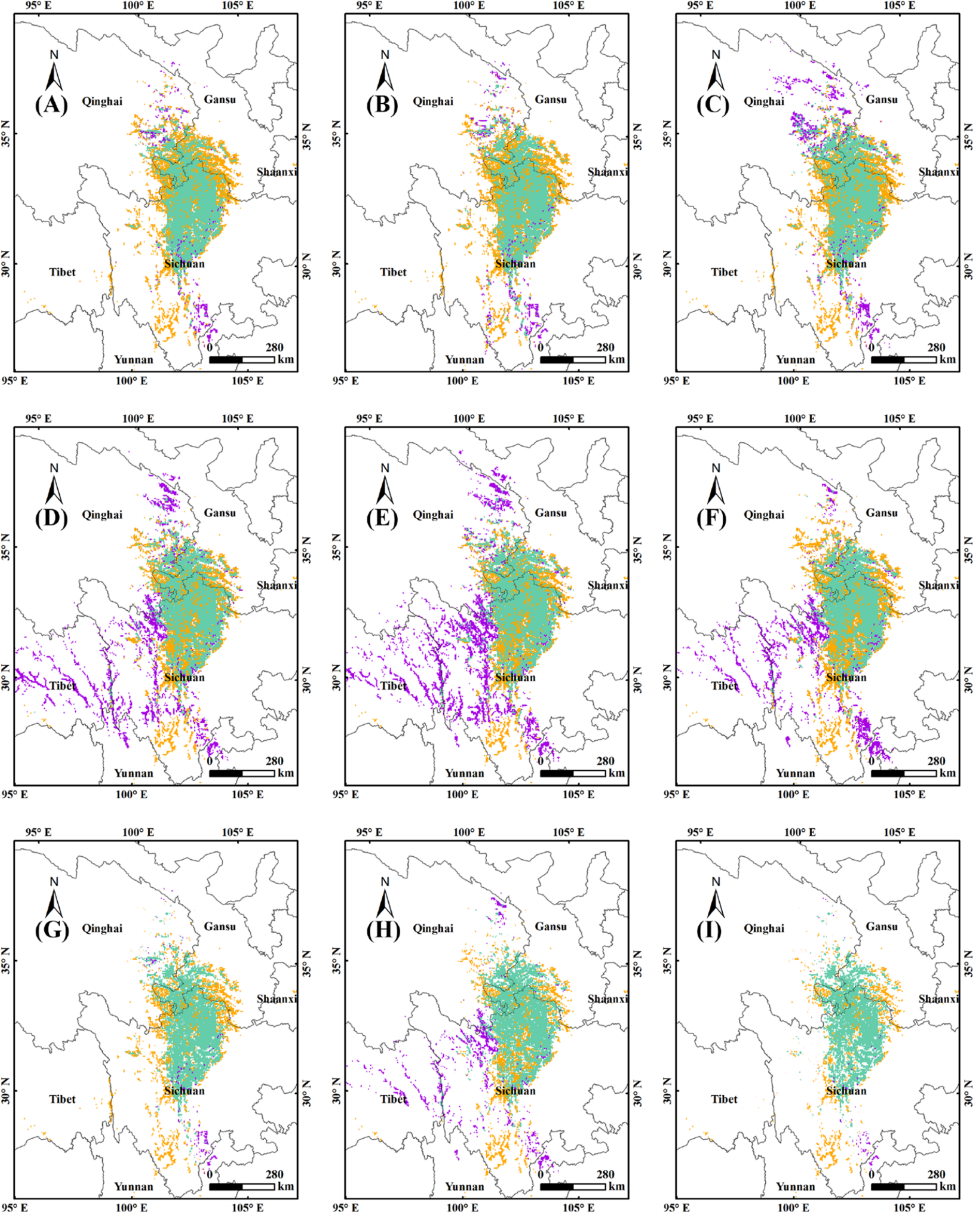
Changes in distribution of suitable habitats in the future with human activities. (**A**): 2050s, SSP1-2.6; (**B**): 2050s, SSP2-4.5; (**C**): 2050s, SSP5-8.5; (**D**): 2090s, SSP1-2.6; (**E**): 2090s, SSP2-4.5; (**F**): 2090s, SSP5-8.5. MaxEnt v3.4.4: https://biodiversityinformatics.amnh.org/open_source/maxent/, ArcGIS v10.0: https://www.arcgis.com/.

Figure 8 showed the stable, expand and shrink area of the suitable habitats in 2050s and 2090s with human activities compared with current situation. In 2050s, the proportion of the stable habitats to the current was 65.15% (SSP1-2.6), 67.06% (SSP2-4.5) and 70.86% (SSP5-8.5) respectively (Fig. 8A,B,C), while in 2090s it was 71.18% (SSP1-2.6), 73.43% (SSP2-4.5) and 67.38% (SSP5-8.5) respectively (Fig. 8D,E,F). The proportion of the shrink habitats to the current was 34.85% (SSP1-2.6), 34.84% (SSP2-4.5) and 32.94% (SSP5-8.5) respectively in 2050s (Fig. 8A,B,C), while in 2090s it was 28.82% (SSP1-2.6), 26.57% (SSP2-4.5) and 32.62% (SSP5-8.5) (Fig. 8D,E,F). The proportion of the expand habitats to the future was 5.58% (SSP1-2.6), 5.88% (SSP2-4.5) and 10.95% (SSP5-8.5) respectively in 2050s respectively (Fig. 8A,B,C), while in 2090s it was 24.56% (SSP1-2.6), 29.3% (SSP2-4.5) and 21.4% (SSP5-8.5) respectively (Fig. 8D,E,F). By comparison, the geographical scope of the stable habitat was somewhat smaller than that without human activities, but the administrative coverage was basically the same. the proportion of the expand habitat tended to decrease with human activities, while the proportion of shrink habitat tended to increase. By overlaying layers in ArcGIS, the suitability change of the suitable habitat in 2050s and 2090s was obtained (Fig. 8G,H,I).

### Centroid variations of the suitable habitats under climate change scenarios

**Figure 9:**
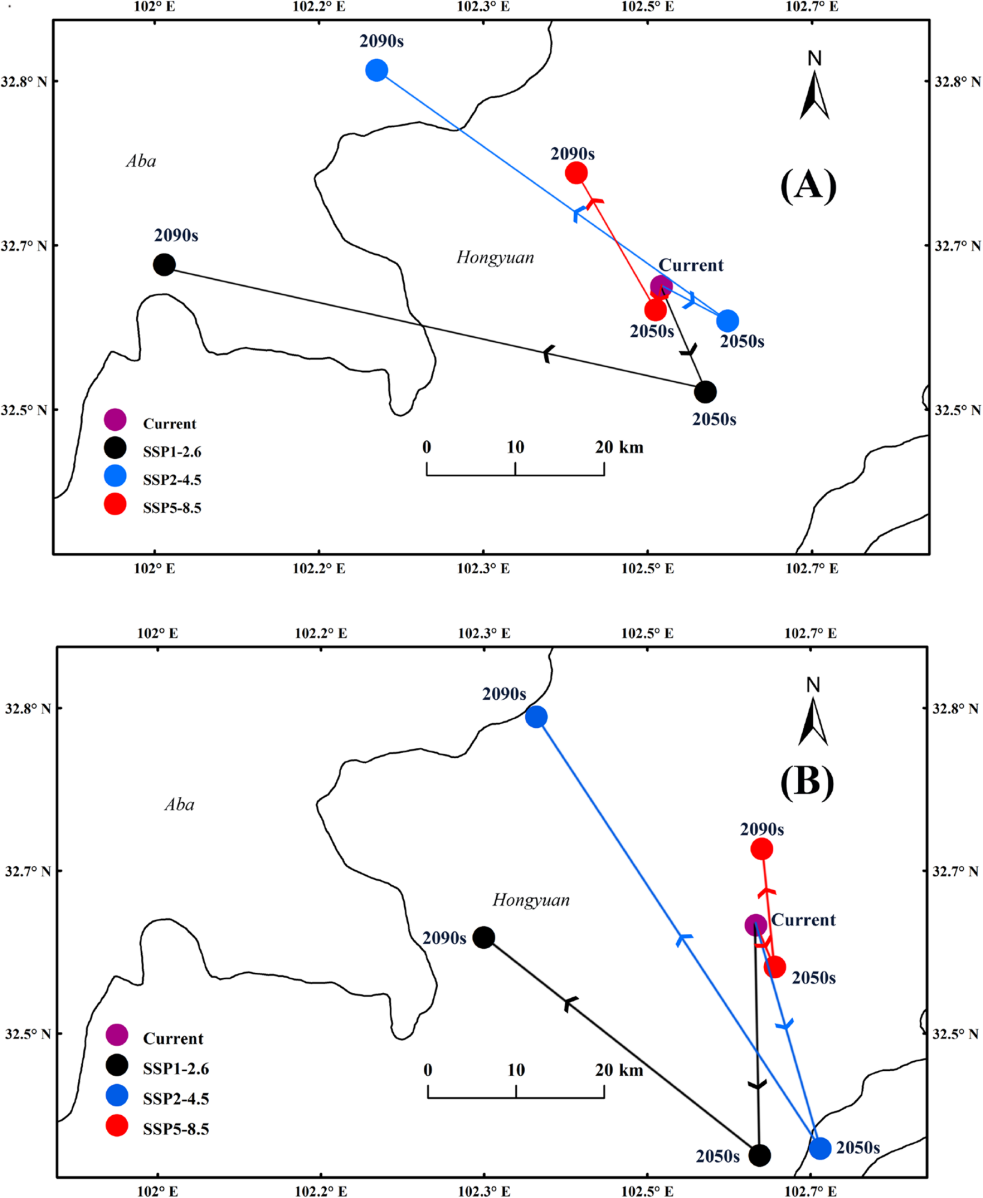
Variations of the centroids of the suitable habitats under climate change scenarios without human activities (**A**) and with human activities (**B**). ArcGIS v10.0: https://www.arcgis.com/.

Without human activities (Fig. 9A), under SSP1-2.6, the centroid of the highly suitable habitats would move 11.62 km from Hongyuan (Current/32.62°N, 102.51°E) to southeast to Hongyuan (2050s/32.52°N, 102.56°E), then 56.4 km to northwest to Aba (2090s/32.65°N, 102.01°E). By 2090s, the centroid would be displaced 50.48 km to the northwest. Under SSP2-4.5, the centroid would move 7.58 km from Hongyuan (Current/32.62°N, 102.51°E) to Hongyuan (2050s/32.59°N, 102.58°E), then 43.76 km to northwest to Aba (2090s/32.84°N, 102.23°E). By 2090s, the centroid would generally displace 36.28 km to the northwest. Under SSP5-8.5, the centroid of the highly suitable habitats would move 2.47 km from Hongyuan (Current/32.62°N, 102.51°E) to southwest to Hongyuan (2050s/32.6°N, 102.5°E), then 16.07 km to northwest to Hongyuan (2090s/32.74°N, 102.43°E). By 2090s, the centroid would generally displace 14.4 km to the northwest (Fig. 9A).

With human activities (Fig. 9B), under SSP1-2.6, the centroid of the highly suitable habitats would displace 23.59 km from Hongyuan (Current/32.61°N, 102.61°E) to southeast to Hongyuan (2050s/32.37°N, 102.62°E), then 35.97 km to northwest to Hongyuan (2090s/32.59°N, 102.33°E). By 2090s, the centroid would displace 27.84 km to the southwest. Under SSP2-4.5, the centroid would move 23.82 km from Hongyuan (Current/32.61°N, 102.61°E) to southeast to Songpan (2050s/32.38°N, 102.68°E), then 52.91 km to northwest to Hongyuan (2090s/32.82°N, 102.38°E). By 2090s, the centroid may displace 30.98 km to the northwest. The centroid of the highly suitable habitats would move 4.69 km from Hongyuan (Current/32.61°N, 102.61°E) to southwest to Hongyuan (2050s/32.56°N, 102.63°E) under SSP5-8.5, then 12.18 km to northwest to Hongyuan (2090s/32.69°N, 102.62°E). By 2090s, the centroid may generally displace 7.84 km to the northeast (Fig. 9B).

### Evaluation of models

Without human activities, based on the training data and test data of the MaxEnt under the current situation, the AUC values were 0.987 ± 0.001 and 0.983 ± 0.013 respectively. Under future climate scenarios, the AUC values for training data were 0.986 ± 0.001–0.988 ± 0.001, while for the test data were 0.979 ± 0.021–0.984 ± 0.006 (Table 3).

**Table 3:** AUC values of models.

Scenario	With human activities		Without human activities	
	Training data	Test data	Training data	Test data
Current	0.987 ± 0.001	0.982 ± 0.015	0.987 ± 0.001	0.983 ± 0.013
2050s, SSP1-2.6	0.987 ± 0.001	0.980 ± 0.015	0.987 ± 0.001	0.980 ± 0.016
2050s, SSP2-4.5	0.988 ± 0.001	0.982 ± 0.012	0.986 ± 0.001	0.979 ± 0.021
2050s, SSP5-8.5	0.986 ± 0.001	0.980 ± 0.013	0.987 ± 0.001	0.982 ± 0.014
2090s, SSP1-2.6	0.988 ± 0.001	0.982 ± 0.009	0.988 ± 0.000	0.984 ± 0.006
2090s, SSP2-4.5	0.989 ± 0.000	0.984 ± 0.004	0.986 ± 0.001	0.983 ± 0.007
2090s, SSP5-8.5	0.989 ± 0.000	0.984 ± 0.006	0.988 ± 0.001	0.984 ± 0.007

With human activities, the AUC values of the training data and test data of the MaxEnt model under current conditions were 0.987 ± 0.001 and 0.982 ± 0.015 respectively. Under climate change scenarios, the AUC values of the training data were 0.986 ± 0.001–0.989 ± 0.000, and that of the test data were 0.980 ± 0.015–0.984 ± 0.006 (Table 3).

## Discussion

As a comprehensive environmental factor that affects plant growth and distribution, altitude largely controls the changes in other environmental factors, such as temperature, precipitation, and light intensity, which exhibit regular changes with altitude^26–29^. Altitude is an important factor affecting Fritillaria plants. Jiang et al. found that the dominant factors related to the growth of wild plants of *Fritillaria cirrhosa* are altitude, precipitation in September, precipitation in November, and vegetation type. Zhao et al. demonstrated through simulation that that although the dominant factors affecting the habitat suitability of *F. cirrhosa*, *F.* *unibracteata*, *Fritillaria przewalskii*, and *Fritillaria delavayi* are different, but altitude plays an important role in all species^24^. *Fritillaria unibracteata* is a kind of alpine plant, which mainly grows in the high altitude area of 2800–4400 m on the eastern edge of Qinghai Tibet Plateau. In this area, altitude is an important factor controlling the combination form and variation degree of other environmental and biological factors^30,31^. The growth status and survival strategy of plants growing in extreme alpine environment can show adaptability to altitude^32^. By comparing, the percent contribution rate of elevation was 44.54% without human activities and 45.63% with human activities, which were the most important variables under both scenarios. *F. unibracteata* grows and reproduces poorly at low altitudes, while the bulb biomass and total plant biomass decreased as altitude increases in high altitudes^33^. Xu et al. demonstrated that the effects of altitude gradient and life history stage on single leaf area, plant height, and specific leaf area of *F.* *unibracteata* were very significant, and there was obvious interaction^34^. Chen et al. believed that the reproductive organs of *F.* *unibracteata* will be affected by altitude, which is mainly related to snow melting time, temperature, ultraviolet intensity, flower visiting insects^35^. Therefore, altitude has a great restrictive effect on the growth of *F.* *unibracteata*. A response curve revealed that the suitable elevation range was 2879.7–3981.82 m, confirming that altitude plays an important role in *F. unibracteata* distribution. Previous studies have shown that within the altitude range of 2371–3076 m, the growth season length of *F.* *unibracteata* is relatively longer, which is conducive to its accumulation of sufficient nutrients to meet the needs of growth and development^26^.

According to the statistical data, in the high-altitude areas of western Sichuan, southeast Qinghai and southern Gansu, the annual precipitation was about 400–1400 mm, and the temperature annual range was large, which was conducive to the accumulation of nutrients and promote the growth of bulb of *F.* *unibracteata*. Based on the response curve, the suitable range for the min temperature of coldest month was −16.84–4.95 °C, while the average temperature during the coldest month was about −2 °C, which could ensure its safe wintering. For the northwest regions with relatively less rainfall (Xinjiang, Inner Mongolia and western Gansu), or the southern coastal areas with more rainfall, it is not conducive to the accumulation of nutrients of *F.* *unibracteata*, so its distribution in these areas is limited. In this study, the suitable range of main variables calculated by MaxEnt was basically consistent with the ecological environment distributed in the field, that was, high altitude, low temperature, thin air, strong sunshine, clear dry and rainy seasons and large temperature diurnal range^34,35^.

The distribution of species can be limited by climate factors at large scales. However, many studies have proved that the use of climate factors separately will lead to a wider predicted niche. Now the most viewpoints are that even in the suitable area, the high fragmentation of natural habitat caused by human activities will hinder the expansion of species. A study by Xu et al. assessed whether and how human activities have changed the extent of 9701 vascular plants' climatic potential ranges in China. and they found narrow-ranged species showed negative range-filling relationships to these human indicators^36^. Zhang et al.'s simulation showed that the modern suitable area of *Rosa persica* was significantly reduced after the addition of human footprint data^37^. Cao et al. compared the impact of human footprint on the suitability of *Swertia przewalskii*, a unique plant in the Qinghai Tibet Plateau, and revealed that after the introduction of this variable, the potential habitat of *S. przewalskii* species showed a fragmentation trend and the area decreased by 32%^6^. In view of this, in order to more comprehensively reflect the ecological environment of *F.* *unibracteata* and obtain accurate results, we not only selected the commonly used bioclimatic variables, but also supplemented altitude, soil factors and human activities. According to our results, human activities had a high percentage impact on the growth suitability of *F. unibracteata* (3.61%). It can be concluded that *F. unibracteata's* growth, habitat and reproduction may be affected by a series of human activities such as population density, land use, roads and railways as well as construction*.*

Under current condition, the highly suitable habitats of *F. unibracteata* were mostly located in northern Sichuan, southern Gansu and southeastern Qinghai and the total area was 6.64 6.64 × 10^4^ km^2^. Field investigation and literature review showed that there were cultivation bases of *F.* *unibracteata* in Western Sichuan Plateau and southern Qinghai located in the predicted highly suitable habitats^38,39^. Liu et al.'s prediction suggested that the most suitable growth areas of *F.* *unibracteata* with an area of 5.62 × 10^4^ km^2^ were dominantly distributed in western and northern Sichuan, south Gansu and south Qinghai, which is consistent with our result^25^. Wang et al. predicted that the best suitable areas for *F.* *unibracteata* were Aba, Ganzi in Sichuan, and southwest Tibet, while Qinghai and Gansu were unsuitable areas, which was different from our results^23^. Our simulation showed that under the impact of human activities, the area of highly, moderately and poorly suitable habitats decreased by 21.63%, 48.91% and 30.84% respectively. Most people believed that even in areas with suitable climate, the high fragmentation of natural habitats caused by human over use of land would hinder the expansion of species habitats^5,40^. Combined with the prediction results and field observation, we speculated that an important reason for the area reduction of *F.* *unibracteata* was the destruction of land and habitat change caused by local urban expansion and road construction. In addition, animal husbandry was one of the important industries in Qinghai Tibet Plateau, therefore the impact of grazing could not be ignored.

Recently, it was found that the suitable areas of several medicinal plants were decreasing under future climate change scenarios. Ji et al. showed that under RCP 2.6, RCP 4.5, RCP 6.0 and RCP 8.5 scenarios, the total suitable area of *Paris verticillata* in China showed a decreasing trend in 2050s and 2070s^18^. A study by Liu et al. calculated the change in the highly suitable area of Fritillariae Cirrhosae Bulbus under SSP1-2.6, SSP2-4.5 and SSP5-8.5, and found that the highly suitable area decreased in size, and the northwest area would be the geometric center of the total suitable area^41^. Wei et al. confirmed that the most suitable area of *Fritillaria walujewii* will decrease in the middle and south of Yili River Valley and Tacheng area^42^. However, some scholars have reached different conclusions. Guan et al. believed that climate warming can promote the growth of some plants, and the suitable area of *Quercus acutissima* will increase in general under future climate conditions^43^. This study found that under human influences, *F. unibracteata* habitats are likely to decrease significantly in 2050s and 2090s. The size of species distribution areas is an important characteristic of species, and narrow range species often have specific environmental needs and may be more sensitive to habitat changes. Under climate change and intense human activities, the distribution area of narrow range species is likely to shrink, while the distribution area of widely distributed species is likely to expand^44–47^. *F. unibracteata* is a typical narrow niche plant, this may explain the significant reduction in its habitat after adding human activities index as environmental variable.

Under the three scenarios, the centroid of *F.* *unibracteata* would move from the current position to the low latitude area first, and then to the high latitude area. As a typical alpine plant, *F.* *unibracteata* has the characteristics of cold tolerance, humidity preference, fear of high temperature and shade preference^19,48^. We speculated that the change of suitable habitat and the moving trend of centroid in the future were the result of the dual influence of temperature and precipitation. Studies have shown that the increase of regional temperature and precipitation will destroy the habitat of some alpine plants^49,50^. By the 2090s, the increasing trend of temperature and precipitation in the Qinghai Tibet Plateau will be more obvious, and the suitability of the original growth area of *F.* *unibracteata* will be further reduced.

With the development of green and healthy industries, medicinal plant resources have attracted increasing attention. However, the contradiction between the demand for traditional Chinese medicine and the increasing scarcity of resources is becoming increasingly prominent^51,52^. Due to strict habitat requirements and indiscriminate mining, the wild resources of *F.* *unibracteata* have been depleted. Therefore, determining the optimal area for artificial introduction and cultivation of medicinal plants through habitat suitability research is one of the effective methods to fundamentally alleviate the pressure on wild medicinal plant resources. The protection of rare and endangered plant resources is not only the key component of biodiversity protection and sustainable utilization, but also one of the important tasks of nature protection. In recent years, people have gradually realized that the loss of species habitat has become the biggest threat to biodiversity. The destruction of the original habitat by reservoirs, ponds and farmland has resulted in the disappearance of many wild populations of *F. unibracteata* in Qinghai and Sichuan^24,53,54^. *F. unibracteata* is urgently in need of better protection. Our GAP analysis showed that there is a large protection GAP of *F.* *unibracteata* in northern Sichuan, southeastern Qinghai and southwestern Gansu. GAP analysis also showed that Taohe, Siguniangshan, Wolong, Sanjiangyuan, Zoige and other more than ten national nature reserves were located within the highly suitable habitats. Therefore, combined with the distribution characteristics in National Nature Reserves and the current national reserve management policy, we put forward the following suggestions: (1) In the areas where the highly suitable habitats overlaps with the National Nature Reserve, the core protection area of *F.* *unibracteata* will be established to protect its habitat completely, and the core protection area will also play a role in the conservation and radiation of its germplasm resources. (2) Studies revealed that it was feasible to transplant *F.* *unibracteata* artificially to a suitable habitat^48,55^. Therefore, for the moderately and poorly suitable habitats, in the areas with dense population, frequent human activities, which have caused serious harm to the groups and germplasm resources of *F.* *unibracteata*. Depending on the environmental conditions such as altitude, soil, temperature and precipitation, they can be transplanted into reservoirs, farms, and parks suitable for their survival and free of human disturbance so as to achieve the purpose of ex-situ genetic resources protection. (3) In the general control area of the National Nature Reserve, the field distribution survey of *F.* *unibracteata* should be further carried out, and the community survey should be carried out in relevant villages and towns to understand the use of local residents' natural resources in the community. On this basis, local residents are encouraged to understand, support and participate in the protection of *F.* *unibracteata* by carrying out environmental education, science dissemination and seeking alternative livelihoods.

## Methods

### Study area and occurrence records of ***F. unibracteata*** in China

**Figure 10:**
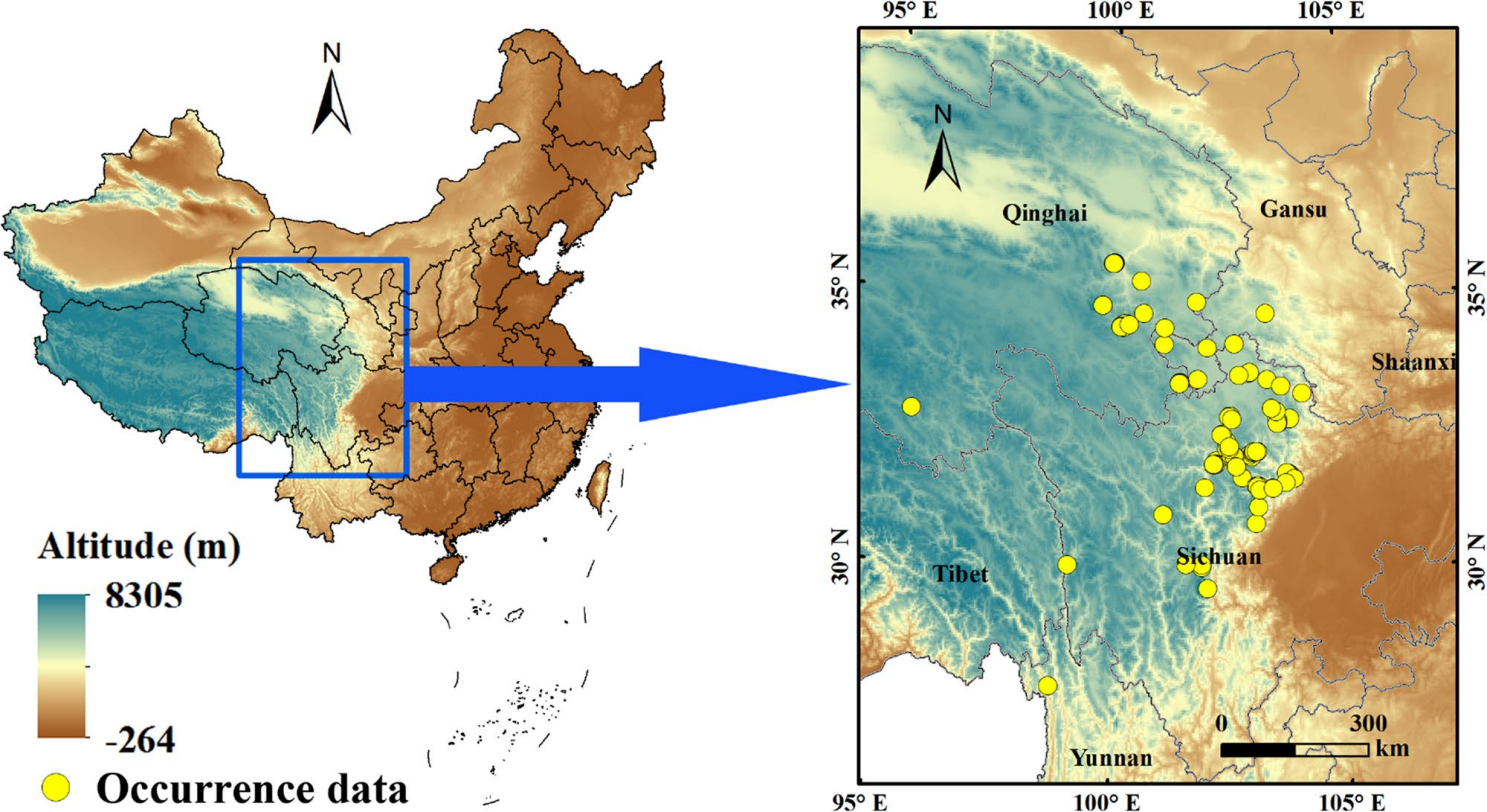
Study area and locations of occurrence records of *F. unibracteata* in China. ArcGIS v10.0: https://www.arcgis.com/.

The study area is predominantly in the eastern edge of the Qinghai Tibet Plateau (Fig. 10), with a large space span, belonging to the plateau marine climate and continental cold dry climate. The air in this area is thin, the annual average temperature is mostly 7 ~ 10 °C, the highest temperature can reach 30 ℃, and the lowest temperature is as low as −20 ~−30 °C. Seasonal frozen layer is distributed in the area above 4000 m, with the maximum thickness of 50 cm. The vegetation is mainly alpine meadow and the overall vegetation coverage is generally low. There is almost no vegetation in the area above 4500 m, with obvious vertical zoning^56^.

Occurrence data of *F.* *unibracteata* were were acquired from the National Specimen Information Infrastructure (NSII, http://www.nsii.org.cn/), the Chinese Virtual Herbarium (CBV, https://www.cvh.ac.cn/), the Global Biodiversity Information Facility (GBIF, https://www.gbif.org/), field survey (Our field survey only recorded the coordinates of occurrence data and did not involve field sampling), and literature. *F. unibracteata* distribution records were processed according to a literature review (Wang et al. 2019; Liu et al. 2021). The first step included using Baidu's coordinate picking system (https://api.map.baidu.com/lbsapi/getpoint) to determine longitudes and latitudes accurate to the town level. The second step involved using Microsoft Excel (2010) to remove duplicate records. Third, each point was measured with respect to its cell grid center, and the nearest point was selected. To establish MaxEnt, 123 distribution points of *F. unibracteata* were retained after the above procedures (Fig. 10).

### Environmental variables

Five independent environmental variables were included in this study (Table S1). Eliminating the influence of multicollinearity was important for simulation, so Pearson correlation coefficient was adopted^57,58^. We first calculated the percent contribution rate of 19 variables using MaxEnt, and we retained the variables with higher percent contribution rates (Table S2). Following that, SPSS was used to analyze Pearson's coefficients between two variables with percent contributions greater than 0 for 123 *F. unibracteata* occurrences. Thirdly, the higher coefficient was retained if the percentage contribution of the variables with the absolute value of the coefficient exceeded 0.85 (Table S3). The prediction model for *F. unibracteata* included 15 variables in addition to elevation (Table 4).

**Table 4:** Environmental variables used to predict the potential geographic distribution of *F.* *unibracteata.*

Code	Variable	Resolution	Unit
Bio2	Mean diurnal range	2.5’	°C
Bio3	Isothermality	2.5’	/
Bio6	Min temperature of coldest month	2.5’	°C
Bio7	Temperature annual range	2.5’	°C
Bio10	Mean temperature of warmest quarter	2.5’	°C
Bio12	Annual precipitation	2.5’	mm
Bio14	Precipitation of warmest quarter	2.5’	mm
El	Elevation	2.5’	m
Aspect	Aspect	2.5’	°
PH	Potential of hydrogen	2.5’	/
T-C	Organic carbon pool topsoil	2.5’	%
T-sand	Topsoil sand fraction	2.5’	%
Depth	Reference soil depth	2.5’	m
UV-B3	Mean UV-B of Highest Month	2.5’	kJ/m^2^
Hf	Human footprint index	2.5’	/

### Modelling process

MaxEnt software operation procedure are presented as: (1) Occurrence data of *F. unibracteata* were imported into the "sample" and "environmental layer" data boxes of MaxEnt software (V3.4.4) in CSV and ASC formats (including bioclimatic variables, soil data, elevation and human footprint). (2) "Create response curves" and "Do jackknife to measure variable importance" were selected respectively to analyze the relationship between variables and presence probability of *F.* *unibracteata* and measure the importance of variables. (3) In the initial model, "Random test percentage" was set to 25%, while in the reconstructed model, "random seed" was selected, and the "replicates" was set to 10^12,59^. An area value under the ROC curve, such as AUC value ranges from 0.5 to 1, is a highly recognized diagnostic test evaluation index^60,61^.

### Supplementary Information

Supplementary Information 1.

Supplementary Information 2.

Supplementary Information 3.

## Data Availability

The data that support the findings of this study are openly available in the Science Data Bank at https://www.doi.org/10.57760/sciencedb.06988.
