# Supplementary material for: Effects of climate warming and human activities on the distribution patterns of Fritillaria unibracteata in eastern Qinghai-Tibetan Plateau
Source: Sci Rep. 2023 Sep 22;13:15770. doi: 10.1038/s41598-023-42988-0 (PMC10516939; doi:10.1038/s41598-023-42988-0)
Supplement: Supplementary file 1 — Supplementary Information 1. [file 41598_2023_42988_MOESM1_ESM.docx]

**Table S1. 26 environmental variables used in this study**

|  | Variables and description | Unit |
| --- | --- | --- |
| Bio1 | Annual Mean Temperature | ℃ |
| Bio2 | Mean Diurnal Range(Mean of monthly (max temp - min temp) | ℃ |
| Bio3 | Isothermality (BIO2/BIO7) (* 100) | / |
| Bio4 | Temperature Seasonality (standard deviation *100) | ℃ |
| Bio5 | Max Temperature of Warmest Month | ℃ |
| Bio6 | Min Temperature of Coldest Month | ℃ |
| Bio7 | Temperature Annual Range (BIO5-BIO6) | ℃ |
| Bio8 | Mean Temperature of Wettest Quarter | ℃ |
| Bio9 | Mean Temperature of Driest Quarter | ℃ |
| Bio10 | Mean Temperature of Warmest Quarter | ℃ |
| Bio11 | Mean Temperature of Coldest Quarter | ℃ |
| Bio12 | Annual Precipitation | mm |
| Bio13 | Precipitation of Wettest Month | mm |
| Bio14 | Precipitation of Driest Month | mm |
| Bio15 | Precipitation Seasonality (Coefficient of Variation) | / |
| Bio16 | Precipitation of Wettest Quarter | mm |
| Bio17 | Precipitation of Driest Quarter | mm |
| Bio18 | Precipitation of Warmest Quarter | mm |
| Bio19 | Precipitation of Coldest Quarter | mm |
| El | Elevation | m |
| Aspect | Aspect | ° |
| PH | Potential of hydrogen | / |
| T-C | Organic carbon pool topsoil | % |
| Depth | Reference soil depth | m |
| UV-B3 | Mean UV-B of Highest Month | kJ/m^2^ |
| Hf | Human footprint index | / |
