# Supplementary material for: Effects of climate warming and human activities on the distribution patterns of Fritillaria unibracteata in eastern Qinghai-Tibetan Plateau
Source: Sci Rep. 2023 Sep 22;13:15770. doi: 10.1038/s41598-023-42988-0 (PMC10516939; doi:10.1038/s41598-023-42988-0)
Supplement: Supplementary file 2 — Supplementary Information 2. [file 41598_2023_42988_MOESM2_ESM.docx]

**Table S2** Percent contribution of 19 bioclimatic variables

| Variable | Percent contribution (%) |
| --- | --- |
| bio10 | 34.7 |
| bio12 | 32.8 |
| bio3 | 13.4 |
| bio7 | 6.4 |
| bio6 | 4.1 |
| bio15 | 2.7 |
| bio5 | 2.2 |
| bio14 | 1.0 |
| bio19 | 0.8 |
| bio16 | 0.6 |
| bio4 | 0.6 |
| bio18 | 0.2 |
| bio2 | 0.2 |
| bio8 | 0.1 |
| bio1 | 0.1 |
| bio17 | 0.0 |
| bio9 | 0.0 |
| bio13 | 0.0 |
| bio11 | 0.0 |
