# Supplementary material for: Effects of climate warming and human activities on the distribution patterns of Fritillaria unibracteata in eastern Qinghai-Tibetan Plateau
Source: Sci Rep. 2023 Sep 22;13:15770. doi: 10.1038/s41598-023-42988-0 (PMC10516939; doi:10.1038/s41598-023-42988-0)
Supplement: Supplementary file 3 — Supplementary Information 3. [file 41598_2023_42988_MOESM3_ESM.docx]

**Table S3. Pairwise Pearson’s correlation coefficients of climatic variables.**

|  | bio1 | bio2 | bio3 | bio4 | bio5 | bio6 | bio7 | bio8 | bio9 | bio10 | bio12 | bio14 | bio15 | bio16 | bio18 |
| --- | --- | --- | --- | --- | --- | --- | --- | --- | --- | --- | --- | --- | --- | --- | --- |
| bio2 | -0.13 |  |  |  |  |  |  |  |  |  |  |  |  |  |  |
| bio3 | 0.42 | 0.56 |  |  |  |  |  |  |  |  |  |  |  |  |  |
| bio4 | -0.58 | 0.44 | -0.44 |  |  |  |  |  |  |  |  |  |  |  |  |
| bio5 | 0.85 | 0.32 | 0.52 | -0.19 |  |  |  |  |  |  |  |  |  |  |  |
| bio6 | 0.90 | -0.45 | 0.31 | -0.80 | 0.60 |  |  |  |  |  |  |  |  |  |  |
| bio7 | -0.42 | 0.81 | 0.02 | 0.85 | 0.08 | -0.74 |  |  |  |  |  |  |  |  |  |
| bio8 | 0.91 | 0.09 | 0.41 | -0.31 | 0.93 | 0.73 | -0.12 |  |  |  |  |  |  |  |  |
| bio9 | 0.97 | -0.21 | 0.48 | -0.73 | 0.77 | 0.95 | -0.56 | 0.83 |  |  |  |  |  |  |  |
| bio10 | 0.96 | -0.06 | 0.33 | -0.38 | 0.90 | 0.82 | -0.27 | 0.95 | 0.89 |  |  |  |  |  |  |
| bio12 | 0.23 | -0.74 | -0.13 | -0.71 | -0.21 | 0.54 | -0.84 | -0.02 | 0.34 | 0.09 |  |  |  |  |  |
| bio14 | 0.17 | -0.66 | -0.25 | -0.47 | -0.19 | 0.42 | -0.67 | -0.03 | 0.25 | 0.06 | 0.75 |  |  |  |  |
| bio15 | -0.24 | 0.71 | 0.19 | 0.60 | 0.19 | -0.50 | 0.78 | 0.01 | -0.32 | -0.14 | -0.88 | -0.75 |  |  |  |
| bio16 | 0.16 | -0.61 | -0.05 | -0.70 | -0.22 | 0.45 | -0.75 | -0.06 | 0.28 | -0.01 | 0.93 | 0.67 | -0.66 |  |  |
| bio18 | 0.15 | -0.60 | -0.04 | -0.69 | -0.22 | 0.44 | -0.73 | -0.06 | 0.27 | -0.02 | 0.92 | 0.67 | -0.64 | 0.99 |  |
| bio19 | 0.11 | -0.64 | -0.25 | -0.46 | -0.25 | 0.37 | -0.67 | -0.10 | 0.21 | 0.00 | 0.76 | 0.96 | -0.82 | 0.65 | 0.65 |
